# Analysis of the Influence of the Outlet Slot on the Distribution of the Product as a Method of Assessing the Quality of Jaw Crusher Operation

**DOI:** 10.3390/ma19112366

**Published:** 2026-06-02

**Authors:** Paweł Ciężkowski, Jarosław Stankiewicz, Sebastian Bąk, Bogumił Chiliński, Jacek Caban

**Affiliations:** 1 Division of Numerical Methods and Intelligent Structures, Faculty of Automotive and Construction Machinery Engineering, Warsaw University of Technology, Ludwika Narbutta 84 Street, 02-524 Warsaw, Poland; bogumil.chilinski@pw.edu.pl; 2 Łukasiewicz—Warsaw Institute of Technology, 6/8 Racjonalizacji St., 02-673 Warsaw, Poland; jaroslaw.stankiewicz@wit.lukasiewicz.gov.pl; 3 Division of Construction Machinery, Faculty of Automotive and Construction Machinery Engineering, Warsaw University of Technology, 84 Narbutta St., 02-524 Warsaw, Poland; sebastian.bak@pw.edu.pl; 4 Faculty of Mechanical Engineering, Lublin University of Technology, 36 Nadbystrzycka St., 20-618 Lublin, Poland; j.caban@pollub.pl

**Keywords:** jaw crusher, mineral processing, parameters estimation, Lorentzian model, particle size distribution, crushing plate wear coefficient, non-sensitive range of outlet slot gap

## Abstract

**Highlights:**

- A measurement system for jaw crusher plate wear assessment is presented.
- The system evaluates wear impact on crushed material characteristics.
- The investigated phenomenon was confirmed by experimental and numerical analyses.
- Combined studies validated the relationship between plate wear and product quality.

**Abstract:**

Producers of aggregates must prove that their products meet required standards. Effective decision-making in crushing processes requires analyzing how crusher operation affects component wear, particularly crushing plates. Their deterioration alters the crusher’s geometry, increasing the closed side setting (CSS) and influencing the final product’s granulation. Monitoring these changes is difficult but essential for ensuring product quality. This study presents a measurement system designed to assess wear in jaw crusher plates and its impact on the crushed material. Since aggregates and crushers vary widely, operational parameters such as wear rate, power consumption, and crushing force also differ, affecting product composition. Therefore, reliable measurement methods are crucial for accurate process evaluation and decision-making. In the analysis of the results, the Lorentzian distribution was used as a flexible tool to mathematically describe changes in the particle size distribution of the ground product. The proposed approach combines granulometric analysis of the crushed product with a Lorentzian model to predict crushing performance. Laboratory experiments confirmed the method’s effectiveness, demonstrating its ability to monitor plate wear and predict resulting product characteristics. In particular, it was observed that a relatively small increase in the CSS (approximately 9.23%) did not result in significant changes in the particle size distribution, indicating the presence of a “non-sensitive” operating range. The results show that systematic wear assessment can improve control over crushing operations and enhance product quality verification.

## 1. Introduction

The primary purpose of this paper is to present the problem of changing the volume of the crusher chamber and to show the relationship between it and the obtained product grain size. When the material is crushed, its grain composition and flow properties change. This study investigated the effect of laboratory jaw crusher parameter settings on the granulometry repose of different types of crushed aggregates.

Crushing is one of the essential technological operations for reducing oversized utility material [1,2,3,4,5,6]. It is important in mineral processing, where the excavated material is converted into a final product or for further treatment [7]. There are several different types of industrial crushers [8,9]. Rational designs of these machines have long been sought, given such criteria as energy [5,10], energy efficiency [11,12], energy size distribution [13,14], energy consumption [15,16,17], performance [18], external loads [19,20], strength and geometric characteristics of products [21,22,23,24,25,26], drive systems, etc. [27]. However, it is a difficult task. It is a consequence of the complexity of crushing processes [28,29,30]. Progress in design is undoubtedly related to the scientific development of the fundamentals of crushing mechanics, which draws on, among others, solid mechanics (numerical methods) [31], impact theory, fracture mechanics, and stochastic theories [32,33,34].

Jaw crushers [35,36,37,38,39] are machines often used as primary crushers [40,41] and sometimes in the second crushing stage. A primary crusher (e.g., a jaw crusher) reduces large rock fragments to a size suitable for further processing, typically in the range of several tens of millimeters, depending on the machine settings and material properties [42,43]. They are characterized by high crushing power, simplicity of design, high operational reliability, and ease of maintenance and repair. Jaw crushers are mainly used for crushing high-strength raw materials [44]. The functional elements of the crushers are two crushing plates (one stationary) [45,46]. The maximum feed grain fed into the crusher should be at most 0.7–0.8 of the width of the crusher inlet. The parameter for adjusting the capacity and grain composition of the processing product for a given raw material is mainly the CSS. The primary cause of crusher interruptions is frictional wear of the crushing components. As a result of the crushing process of natural raw materials, crushing elements are abraded [47,48]. Wear occurs due to the friction between the material and the crushing chamber plates and between pieces of material themselves. The operation of jaw crushers depends on how the moving jaw is driven relative to its working surface. For crushing action to occur, the direction of the jaw’s movement must be perpendicular to its surface. If the swing axis of the moving jaw is located outside the working plane, the force is distributed into a normal force, which causes compression, and a tangential force, which causes wear. Wear is undesirable during the crushing of dry materials—it leads to rapid wear of the crushing chamber components and contributes to dust formation. In the case of wet materials, tangential forces on the surface of the moving jaw cause the material adhering to the jaw to be cleared.

**Figure 1:**
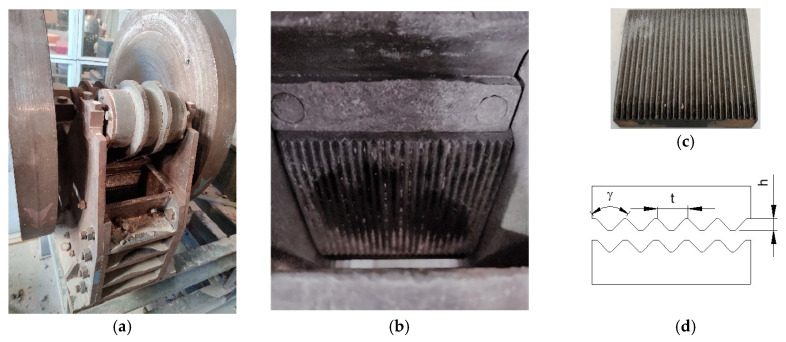
Laboratory stand: (**a**) jaw crusher; (**b**) crusher chamber, top view; (**c**) crushing plate used; (**d**) plate alignment scheme.

Both plates and body armor plates gradually wear [47,49,50,51]. A factor that adversely affects the proper operation of the crusher and limits the life of its other components is uneven wear on the crushing plates. The crushing plates are subjected to intense friction due to the hardness, density, and inhomogeneity of the crushed materials. Abrasive wear is a process of intensive destruction of the teeth of the crushing plates. Plastic deformation occurs, leading to micro-cutting of the teeth and unevenness of these elements (Figure 1). A variety of factors influences the lifetime of comminution plates, i.e., the material from which the plates are made, the physical and chemical properties of the feed, the design of the crushing plate (their cross and longitudinal profile) [20,52,53], the technological system adopted [30,54], crusher kinematics, operational parameters of the crusher (size, outlet slot size, feeding method).

According to previous literature reviews [55], the stationary plate of a crusher exhibits a higher degree of wear compared to the moving plate. However, the wear intensity on working components depends not only on the equipment’s operating conditions but also on the mineralogical properties of the crushed material. Changes in the mineralogical composition of the feedstock can significantly affect the mechanisms and rate of wear on the working surfaces of the crushing plates [56]. Another factor affecting the rate of wear on crusher components is the proper optimization of feed particle size and the geometry of the working jaws. Proper selection of these parameters can reduce wear on working components by up to 50%, thereby increasing crushing efficiency and improving operational economics [57].

Wear on crusher liners alters the geometry of the crushing chamber, significantly impacting the process and product specifications [55]. As wear progresses, changes in crushing conditions and the material’s particle size distribution become apparent [58]. This phenomenon is directly linked to the need to adjust the CSS, as such adjustments compensate for the wear of the working components [59].

A review of the literature indicates that the wear of jaw crusher working plates is asymmetrical, and that the wear mechanisms of the fixed and moving plates differ due to the different kinematics of the material–working surface contact. Lindqvist and Evertsson, in laboratory studies of quartzite crushing, demonstrated that pressure distributions and local grain slippage cause varying degrees of wear on both plates, with the highest loads and most severe wear observed in the lower zone of the crushing chamber, near the discharge gap. The authors also concluded that the wear mechanism cannot be explained solely by classical sliding abrasion, but that the local movement of crushed grains under high pressure plays a significant role [55].

Further studies confirmed that the wear rate of liners is strongly dependent on the outlet slot gap (CSS), rock type, and feed distribution. As the working gap increases, the crushing chamber geometry, pressure distribution, and final product grain size distribution change, affecting both process efficiency and the intensity of wear on the working elements [58].

In material studies, Machado et al. also demonstrated that the stationary and moving plates can exhibit different mass loss values and distinct wear micro-mechanisms [50]. Under the conditions of their experiment, greater wear was observed for the stationary plate, which the authors attributed to the dominance of micro-cutting and micro-grooving. In contrast, micro-indentation played a more significant role for the moving plate. These results confirm that the wear relationship between the two plates depends on operating conditions and is not universal [55].

A review of the literature indicates that wear on the working plates of jaw crushers is uneven. Lindqvist and Evertsson demonstrated that wear intensity in the lower part of the crushing chamber [55], near the discharge gap, can be 2–3 times greater than in the upper part. Machado et al., on the other hand, found that under certain operating conditions, wear on the fixed plate can be 18–25% greater than on the movable plate [50]. Additionally, it was shown that increasing the proportion of grain slip can increase the wear rate by 30–40% [16], and that changes in jaw geometry can reduce local wear by approximately 12–18% [60]. These results confirm that plate wear depends on the chamber geometry, contact conditions, and discharge gap setting.

The service life of jaw crusher liners is strongly dependent on the properties of the feed material, particularly its abrasiveness. Under conditions of heavy material abrasion, the service life of the liners can range from several hundred operating hours, typically 600–900, according to technical data and industrial observations [61]. The literature indicates that jaw crusher liner wear is nonlinear, with a marked acceleration during the final phase of operation. This is associated with progressive degradation of the crushing chamber geometry, increased grain slippage at the material–lining interface, and stress concentration in the discharge gap region [16,55]. Consequently, this leads to a sharp deterioration in the crushing process efficiency and changes in the equipment’s operating conditions, as confirmed by studies on the wear of manganese linings under operating conditions [50].

The efficiency of the rock-crushing process also depends on the shape of the crushing plate surface [10,62,63,64]. The authors [65] studied the effect of crushing process parameters on the quality of broken aggregates in a jaw crusher. They noted that the parameters of the resulting material (grain size, particle shape) vary with the crusher settings used. A study of rock crushing found that rock fineness increases with the LA ratio [23]. There are quite a few papers in the literature showing the influence of the type of crusher used [66,67,68,69,70] or the wear of crushing plates [45,71] on the crushing process parameters [72,73,74].

In industrial practice, one encounters crushing plates with flat or wedge-shaped protrusions [54]. However, it should be noted that the latter, even after a very short period of operation, wears out and operates as flat stamps, which increases the CSS of the crusher during operation.

As mentioned earlier, over time, the crushing plates of the crusher wear out and need to be replaced to maximize crusher efficiency and avoid machine failure or damage. Crushers are already available that maintain a constant CSS [46,75]; the position of the crushing plate can be automatically adjusted during operation. If the CSS is not maintained, undesirable changes in product sizes and/or production issues may occur. According to European standards, e.g., CEN—PREN 13043 [64], the aggregate producer must assess the quality of the raw material produced.

Presented research shows that wear affects the granulometric distribution, like increasing the gap between plates, as described [16,55,76].

Considering the above, it is evident that a key step towards a more rational and efficient rock crushing process is a better understanding of how increasing CSS affects the crushing process, particularly the granulometric composition of the resulting product. The first step is laboratory crushing, which is performed as part of preliminary product quality assessment studies.

One of the essential pieces of information for the crusher user is the grain composition of the product, i.e., information about the share of individual fractions [77]. Since crushers not equipped with an outlet slot compensation system (mainly jaw crushers) experience an increase in the outlet slot dimension as a result of wear and tear, and as a result, change the share of fractions, information essential for the operator is: 1—to estimate the time (weight of crushed material) when it is necessary to correct the outlet slot or replace the crushing plates, 2—to determine the effect of increasing the outlet slot on the product grain composition—this is the main aim of the article. The grain composition of the crushing product changes over time due to the statistical nature of the crushing process. Estimating the weight distribution of individual fractions at a constant outlet slot in the crusher and for a constant feed granularity will allow deducing the significance of decreasing the outlet slot dimension on the change in the weight distribution of product fractions. The effect of the outlet gap on product size distribution is well known; however, the impact of small changes in the gap remains relatively poorly understood in the literature [55,78,79]. A suitable stochastic model that accounts for the influence of the CSS outlet slot dimension and the type of crushing material should be feasible based on experimental laboratory tests.

This article focuses on developing a system to evaluate the effectiveness of jaw-crusher crushing. It aims to deepen understanding of how to achieve optimal crushed product composition in a jaw crusher, more reliably and efficiently, by examining the interaction between the crushed product granulometric composition and the discharge gap size. Both experimental studies and statistical analyses were conducted to control the crusher’s outlet slot size while maintaining the product’s granulometric composition within acceptable limits.

Research assumed the following methodology. The first stage is a comprehensive review of the existing literature, focusing on current research on crushers and the influence of machine parameters on the crushing process. A brief description of the jaw crusher and its role in crushing is also presented. The next stage considers in detail the jaw crusher used in the laboratory studies and describes the research methods applied in the experimental setup, including the types of aggregates studied. After that, the experimental results were presented, illustrating the relationship between the outlet slot size and the product’s grain size distribution. This stage also presents an innovative approach, highlighting the development of a system for testing granulometric parameters in crushers using custom-integrated sensors. Finally, the conclusions are drawn from the studies, suggesting potential improvements in crushing technology and pointing to future directions for the development of monitoring techniques, especially in light of advancements in measuring crusher discharge gap and product granulometric distribution. The results presented in this paper have significant implications for the design and development of jaw-crusher crushing processes.

Machine learning methods, widely used across various engineering fields, are also applied to crusher monitoring [80]. They enable the analysis of operational data, the identification of equipment malfunctions, and the prediction of wear on working components, thereby increasing the reliability and efficiency of the crushing process.

In recent years, there has been growing interest in applying machine learning (ML) and artificial intelligence (AI) to mineral processing and crushing. These methods are used, among other things, to predict grain size distribution, monitor equipment condition, and optimize process parameters, enabling the mapping of complex, nonlinear relationships between operational variables. Modern approaches based on AI and data analysis have demonstrated significant potential to improve process control and predictive maintenance in engineering systems [81,82,83,84,85,86,87].

Despite these advances, models based on interpretable physical relationships remain highly relevant, particularly in crushing processes, where understanding the relationships between machine operating parameters and material behavior is critical. Jaw crushers, as primary crushing equipment, are characterized by complex interactions among the discharge gap (CSS), material properties, and wear of the working components, which directly influence the product particle size distribution.

In this context, the development of highly interpretable mathematical models, such as the Lorenz-based approach used in this study, complements methods based solely on data. This approach enables both a better understanding of the process mechanisms and practical applicability under industrial conditions.

Table 1 presents a specification of the literature studies and a research gap analysis. 

Data analysis indicates that the studies focus on selected aspects of the crushing process but lack final links between particle size distribution and user modeling. This work requires applying the Lorentzian model to describe process changes.

## 2. Materials and Methods

### 2.1. Laboratory Stand

In this work, a strong emphasis was placed on experimental studies, as they provide a measure of the validity of assumptions for theoretical analyses. Operational tests were conducted on a model double-toggle jaw crusher using four rock materials with different physical properties: marble, dolomite, quartzite, and basalt. The tests were carried out in a jaw crusher with a straight jaw motion (Figure 1a). A set of triangular-profile plates with fixed pitch and misaligned teeth was used in the tests (Figure 1b,c). The pitch between the notches was t = 6.5 mm, tooth height h = 4.5 mm, and tooth apex angle γ = 90° (Figure 1d). The test crusher has the following technical parameters: crusher inlet slot dimension 60 mm × 130 mm, outlet slot CCS = from 3 to 8.5 mm, working chamber height 175 mm, moving jaw stroke s = 5 mm, drive shaft speed n = 375 rpm, and motor rated power N_zn_ = 1.5 kW.

The crushing plates used in the study were made of C45 medium-carbon structural steel, with a hardness of approximately 44 HRC. All experiments were conducted using a single set of crushing plates, ensuring consistent material properties throughout the study.

In the analysis, however, the operating parameters were varied, in particular the outlet gap (CSS) and the properties of the feed material, to simulate different system operating conditions and corresponding wear states. This approach allows assessing the impact of operating conditions on the crushing process and product characteristics while keeping the material properties of the working elements constant.

It should be emphasized that the specific influence of the chemical composition, microstructure, and tribological properties of the plate material on wear mechanisms was not the subject of this study.

The triangular profile of the working surface of the crushing plates used in the study, together with the specified tooth spacing, constitutes a standard design solution used in laboratory jaw crushers. This geometry remained unchanged throughout the experiments and was determined by the equipment’s configuration.

According to the literature, the shape of the working surface of jaw crusher liners significantly affects the crushing mechanism and the distribution of wear. Serrated and profiled working surfaces increase material grip efficiency, intensify local contact stresses, and promote the initiation of cracks in the crushed material through the combined effects of compression and shear [16,55].

More recent studies also indicate that tooth geometry and pitch influence local material–coating contact conditions, thereby modifying the contributions of sliding and crushing mechanisms and affecting both the efficiency of the crushing process and the intensity of wear on working elements [48,89]. In particular, it has been demonstrated that changes in the working surface geometry can significantly alter the product size distribution and the location of the zones of maximum wear.

Consequently, the crushing plate geometry used reflects typical operating conditions for jaw crushers and represents a compromise between crushing efficiency and the durability of the working components.

### 2.2. Experimental Methodology

**Figure 2:**
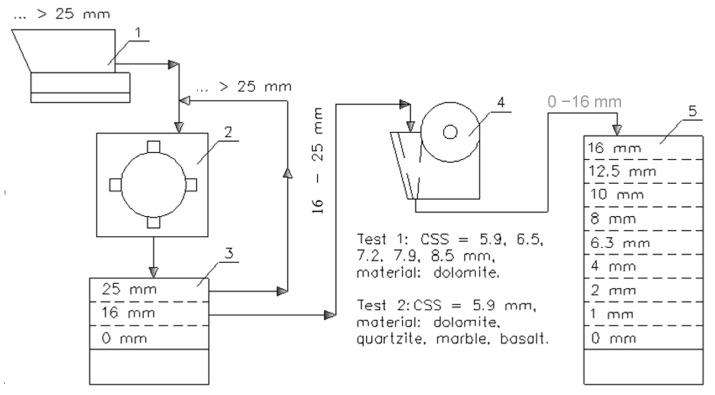
Diagram of the technology of rock processing.

Figure 2 shows the diagram used to test the jaw crusher. The granulometric composition of the test products, as shown in Table 2, compared with the feed composition, provides an approximate overview of the degree of rock crushing in the jaw crusher.

**Figure 3:**
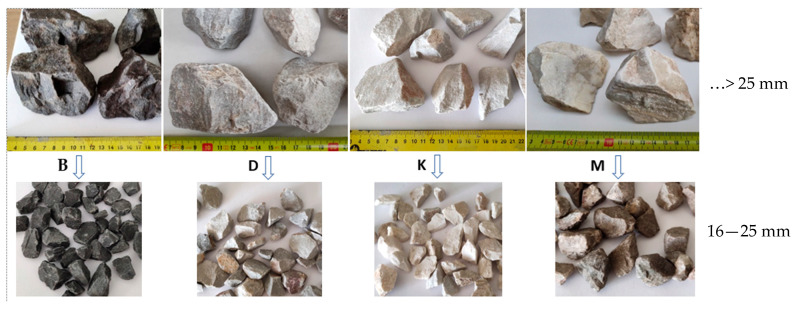
Sample grains before (upper row) and after (bottom row) the crushing process in the impact crusher. B—basalt, D—dolomite, K—quartzite, and M—marble.

The research was carried out in two stages (Figure 2). In the first stage, D dolomite feed (Figure 3) was used to investigate the effect of CSS outlet slot size on grain composition and fineness. These samples were initially crushed in an impact crusher (number 2 in Figure 2) to a grain size of 16–25 mm (3) and then divided into equal portions weighing about 2 kg. The impact crusher served as a preparatory device, pre-crushing the material and producing a 16–25 mm fraction. The raw materials were pre-crushed using an impact crusher to a particle size of 16–25 mm. This range was selected in accordance with the material feeding requirements and the operating parameters of the jaw crusher used in the subsequent stage of the research. The selected fraction ensured stable material feeding, reduced the risk of inlet blockage, and allowed the crushing process to be conducted under more uniform conditions. Furthermore, reducing the variation in the grain size of the feed material improved the reproducibility of the experimental results obtained. Grains larger than 25 mm were re-crushed and screened to ensure that the jaw crusher received material of the appropriate particle size for further testing. The amount thus prepared constituted a single sample and was crushed in a jaw crusher (4). A series of tests for setting one outlet slot was conducted for at least 10 tests. The results presented in this study are the average values of the measurements obtained. An analysis of the data distribution revealed little variation between replicates, confirming the stability of the experimental process and the absence of significant deviations that would affect the interpretation of the relationships obtained. Individual samples were crushed at CSS outlet slot sizes of 5.9, 6.5, 7.2, 7.9, and 8.5 mm. The study focuses on the effect of CSS, assuming constant operating conditions of the laboratory crusher. It should be noted that the outlet gap (CSS) values used, ranging from 5.9 to 8.5 mm, correspond to the operating conditions of a laboratory-scale crusher. In the first stage of the dolomite study, five CSS values were used, spanning the typical operating range of a jaw crusher. The intervals between successive settings were selected to allow analysis of the effect of a gradual change in the discharge gap on the crushing process efficiency and the product particle size distribution. The adopted set of CSS values also served as a simplified representation of the wear of the working jaws, which causes a gradual increase in the discharge gap during operation. The range of tested values was determined based on the equipment’s technical parameters, preliminary tests, and literature data. Unlike industrial crushers used in aggregate processing, where CSS values are significantly higher, laboratory studies employ smaller settings to ensure controlled experimental conditions and enable detailed analysis of the influence of process parameters on crushing and the product particle size distribution. The CSS range used does not directly reflect industrial conditions. Still, it allows for the analysis of fundamental relationships that can be utilized in further modeling and interpretation of industrial-scale processes. The grain composition and degree of fineness were evaluated using a sieve analysis (5) based on outlet slot size and the feed. The analysis was performed using the screening method in accordance with EN 933-1:2000 [90], thereby ensuring the repeatability and comparability of the results. Analysis of the impact of alternative sieving methods was not the subject of this study and may be the subject of further research. In the second stage of the study, the following materials were used as feedstock: B—basalt, D—dolomite, K—quartzite, and M—marble, to determine the effect of material type on the product’s grain composition. All rocks originated in Poland; the dolomite came from the “Jaźwica” deposit, while the marble was of the “White Marianna” variety. The densities of the tested rocks were approximately: basalt—2.9 g/cm^3^, quartzite—2.65 g/cm^3^, dolomite—2.85 g/cm^3^, and marble—2.65 g/cm^3^. To further characterize the studied rocks, data were supplemented with uniaxial compressive strength (UCS). This parameter is a fundamental indicator of rock material mechanical properties and can influence their behavior during crushing. The uniaxial compressive strength of the tested rocks was approximately: basalt—220 MPa, quartzite—180 MPa, dolomite—97 MPa, and marble—87 MPa. The jaw crusher tests were conducted with a CSS of 5.9 mm.

Exemplary photos of basalt, dolomite, quartzite, and marble grains before and after the crushing process in an impact crusher are shown in Figure 3.

Material samples for particle size analysis were taken from the final product under steady-state operating conditions, assuming they were representative of the entire material stream. To minimize sampling errors, multiple samples were collected from different sections of the stream and homogenized before particle size analysis.

This approach allows for an approximate representation of random sampling conditions and minimizes the impact of local material heterogeneities. The good repeatability of the results indicates that the samples are sufficiently representative under the test conditions.

It should be noted, however, that under industrial conditions, full representativeness of sampling requires the use of standardized methods and dedicated sampling systems. A detailed statistical analysis of the sampling characteristics was not the subject of this study and constitutes a direction for further research.

### 2.3. Product Grain Size, Grain Distribution

**Figure 4:**
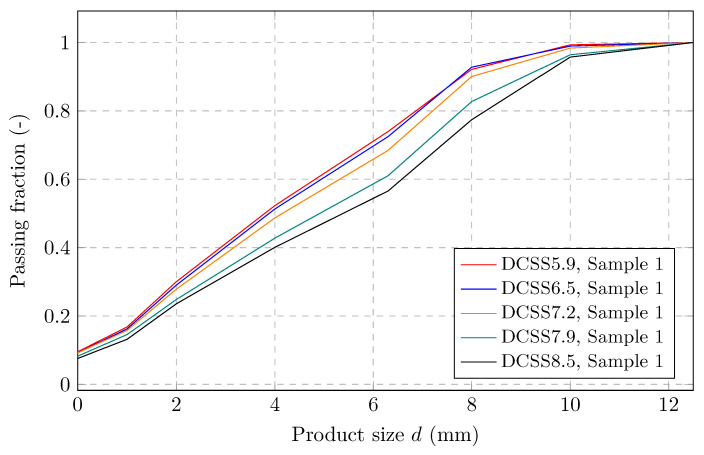
The effect of the CSS discharge gap of a jaw crusher on the particle size distribution of crushed dolomite. Example designation: DCSS5.9, where D stands for dolomite and CSS = 5.9 mm.

The collection of grains after crushing is challenging to describe mathematically. Grains vary in shape and dimensions even within the same selected group. There are many methods for determining the surface area of grains [91,92]. An additional issue is the analysis of grain shape. The distinction between regular (cubic), elongated, flat, and needle grains is made. A grain distribution curve describes the dimensions of a group (set) of product grains. It shows the relationship between grain size and its frequency of occurrence within a given grain group. Granulometric analysis is an essential parameter used to evaluate the correctness of many technological processes. Its results are an integral part of assessing and optimizing the operating parameters of many devices, including crushers. Analysis of grain group dimensions, especially those resulting from the crushing process, provides information on the expected quantities of the product in grain fractions with given boundary dimensions. Such information is the leading information from the point of view of the designer and manufacturer conducting the crushing process. The samples were sieved according to the EN 933-1:2000 [90]. The screening took 10 min using a Morek Multiserw Type 1 PzE-3e laboratory sieve shaker, a time selected based on the device’s operational parameters and preliminary tests, which indicated that this duration is sufficient to achieve stable sieving conditions and that further extension of sieving time does not significantly affect the particle size distribution results. The results presented in Table 2 are based on a single series of 10 replicates and presents representative sieve analysis results for dolomite at a CSS of 8.5 mm. All CSS levels were investigated under identical experimental conditions, with 10 replicates per condition, ensuring methodological consistency and comparability of results. Figure 4 reports mean values obtained from five independent test series (10 replicates each), which were used to ensure statistical reliability of the analysis. The complete dataset was fully used for quantitative evaluation and trend interpretation, although only representative results are presented in the main text for clarity and conciseness. This approach does not affect the validity of the conclusions, which consistently demonstrate a clear effect of CSS variation on the product’s particle size distribution.

**Table 2:** Results of the sieve analysis for 10 replicates—dolomite CSS = 8.5 mm.

Dimensions of the Sieve [mm]	Residue on Sieve (%)										Mean (%)	Standard Deviation (%)
	Test 1	Test 2	Test 3	Test 4	Test 5	Test 6	Test7	Test 8	Test 9	Test 10		
0	7.57	6.97	7.74	7.04	7.27	7.55	7.26	6.91	7.14	6.84	7.36	5.9
1	5.64	5.34	5.88	5.34	5.54	5.82	5.74	5.34	5.50	5.04	5.59	4.9
2	10.38	10.07	10.62	9.83	10.52	10.58	10.55	9.84	9.97	9.38	10.33	7.9
4	16.50	15.25	17.61	16.29	16.08	16.72	17.19	16.20	16.50	15.92	16.41	12.8
6.3	16.48	14.86	15.66	16.31	15.19	15.21	15.10	14.46	16.93	14.90	15.62	14.9
8	20.85	21.53	20.09	23.02	22.38	20.09	19.74	21.41	21.73	21.73	21.33	19.8
10	18.33	19.04	17.11	16.41	17.23	17.71	18.56	18.99	16.84	18.56	17.64	17.9
12.5	4.26	6.93	5.29	5.78	5.79	6.32	5.86	6.86	5.39	7.62	5.73	18.2

The standard deviation values presented in Table 2 refer to the mass fraction of grain fractions and provide a measure of the variability of results obtained in experimental replicates. Small standard deviation values indicate good repeatability of measurements and stable experimental conditions.

The first stage of analysis concerns the influence of the CSS on product distribution. As shown in Figure 4, increasing the CSS outlet slot by 9.23% did not significantly alter the obtained granulometric composition, which is a valuable observation. In turn, subsequent increases in the outlet slot size alter the product composition, disrupting the adopted technological process parameters and requiring adjustments to the outlet slot settings to maintain the assumed product parameters.

**Figure 5:**
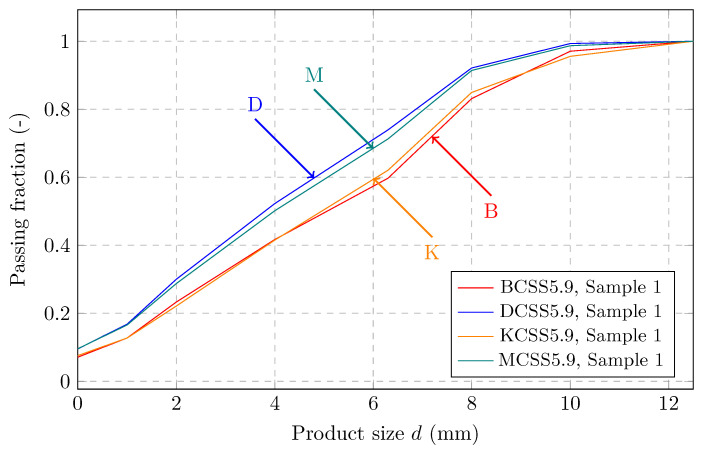
Granulometric distribution of different materials: B—basalt, D—dolomite, K—quartzite, and M—marble for CSS = 5.9 mm.

The next stage of the investigation was the analysis of the material’s influence on product size. Four rock types were tested and divided into two groups: harder and softer. Product distributions were demonstrated in Figure 5.

The impact of the material type is visible in Figure 5. Harder materials are located above the softer materials. There is a visible gap between hard and soft materials. The assessment condition is the material’s compression stress or hardness.

Preliminary analysis of the results on the example of 10 samples (Table 2) allowed us to conclude that the grain compositions of the crushing products obtained by individual crushing for the same CSS outlet slot show only slight differences (within the limits of the error of analysis), and therefore, for further consideration, the averaged granulometric distributions were adopted by comparing the obtained products for different slots. Sieve analysis enabled the determination of the granulometric composition of the product for the jaw crusher outlet slot settings (Test 1 in Figure 2) and the four tested rock types (Test 2 in Figure 2). The results show that gradually increasing the outlet slot size decreases the proportion of the product’s fine fraction.

In the second stage of the research, based on the results for each grain class, a granulometric summary distribution of dolomite, basalt, quartzite, and marble samples was created for the outlet slot with CSS = 5.9 mm. It should be noted that the grain distribution curves obtained for dolomite and marble, as well as quartzite and basalt, have a similar character. For materials of lower strength (dolomite, marble), a more significant amount of fine fraction was obtained in the crushing product.

In research on the crushing process of brittle materials, one of the main tasks is to establish relationships regarding changes in the grain composition of the feed during the considered process. Many methods of varying complexity have been developed to determine the size distribution of particulates [62,93,94,95]. However, it is difficult to select a single general approach [96,97,98,99,100]. The most popular are the Schumann power distribution [40,101,102], the Rosin–Rammler–Sperling distribution [32,88], the Kolmogorov distribution [103], and the Gaussian distribution [104,105,106,107,108]. The mathematical description of grain distribution curves is still under development, so new models are needed to address the current lack of scientific progress. The necessity of correctly determining the dimensions of grains replacing feed and product grains of different sizes is generally recognized.

### 2.4. Block Diagram of the Methodology Used

**Figure 6:**
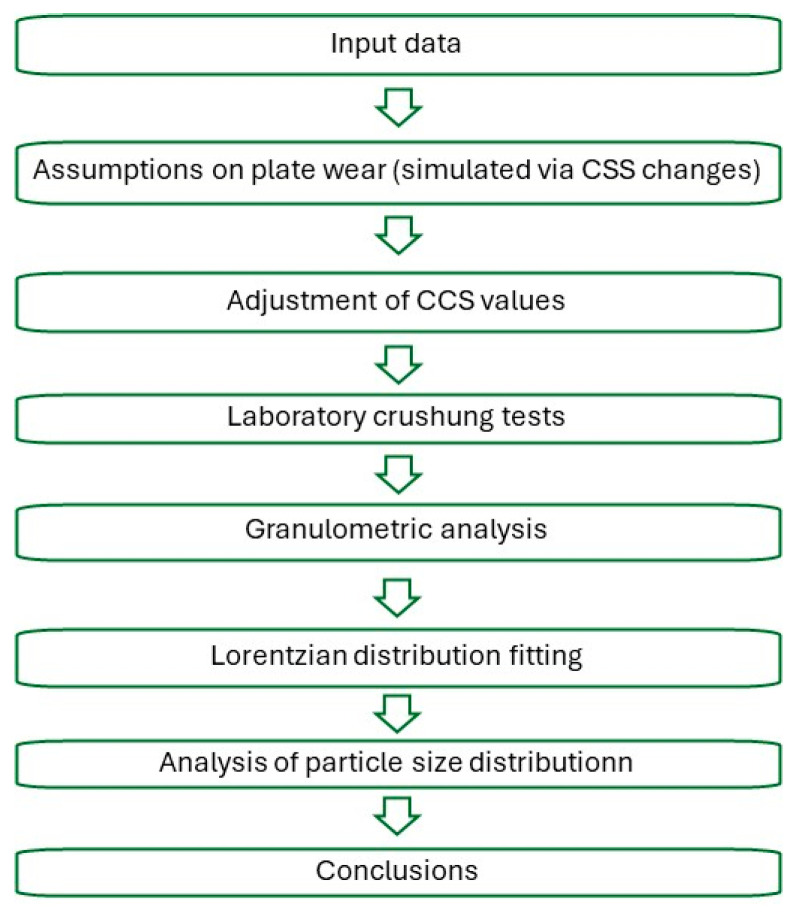
Flow chart of the methodology for analyzing the effect of CSS changes on particle size distribution.

To enhance clarity, Figure 6 presents a flowchart of the research methodology. It illustrates the subsequent stages of the analysis, starting with input data and assumptions about changes in the CSS outlet gap to simulate crushing plate wear, followed by crushing tests under laboratory conditions, and finally grain size analysis of the resulting product. 

In this study, wear of working elements was not measured directly but rather modeled through controlled changes in the CSS value. This approach enables the analysis of the impact of changes in crusher geometry on the product’s grain size distribution in a repeatable, controlled manner.

## 3. Results

### 3.1. Modeling Grain Size Distributions

The analysis of the fit of the Gaussian and Lorentzian models presented in this paper was conducted based on data obtained for dolomite, which was chosen as the reference material due to the high reproducibility and completeness of the experimental data. This allowed for an unambiguous assessment of the fit quality and an interpretation of the physical significance of the model parameters.

It should be emphasized, however, that the proposed modeling approach is general and can be applied to other rock materials. A preliminary qualitative analysis indicates that the observed relationships among the other materials studied are similar in nature. However, the model parameters vary with the mechanical and structural properties of the rocks.

Full validation of the model for various types of materials, based on systematic comparative studies, is beyond the scope of this work and constitutes a direction for further research.

Least-squares determined the Lorentz model parameters, and their uncertainties were estimated from the covariance matrix obtained during the fit. The resulting parameter standard errors reflect the impact of the experimental data’s variability on the stability of the estimates.

The main sources of uncertainty include experimental variability, limited data points, and model simplifications. The obtained uncertainty values serve as a measure of the model’s sensitivity to changes in input data and of the quality of the fit.

It should be noted, however, that this analysis is simplified and does not include a full uncertainty propagation. A more detailed analysis that accounts for all sources of measurement and modeling errors is the focus of further research.

The mathematical model was proposed according to the methodology proposed in the paper’s assumptions. Two models of grain distribution were proposed—the Gaussian and Lorentzian models. The following formula describes the first one:

(1) $$f\left(d,A,\mu ,\sigma \right)=\frac{A}{\sqrt{2\pi }\sigma }e^{-\frac{({d-\mu )}^{2}}{2\sigma^{2}}}$$

where f—granulometric distribution, μ—center (first moment), A—the maximal value of distribution, σ—RMS, d—independent variable (in this paper: grain size).

The second model (Lorentzian) has the following form:

(2) $$f\left(d,A,\mu ,\sigma \right)=\frac{A}{\pi }\cdot \frac{\sigma }{\sigma^{2}+{\left(d-\mu \right)}^{2}}$$

where f—granulometric distribution, mu—median, A—maximal value of the distribution, sigma—median absolute deviation, d—independent variable (in this paper: grain size).

The Gaussian distribution parameters have a clear statistical interpretation in terms of particle size distribution, with their values directly corresponding to the mean size and deviation of particle sizes. The Lorentzian function should be treated primarily as a phenomenological peak-shape model, as its parameters do not have a direct physical interpretation in terms of particle size distribution. Nevertheless, the Lorentzian model can still be successfully applied for fitting purposes, despite the lack of a straightforward physical meaning of its parameters.

**Figure 7:**
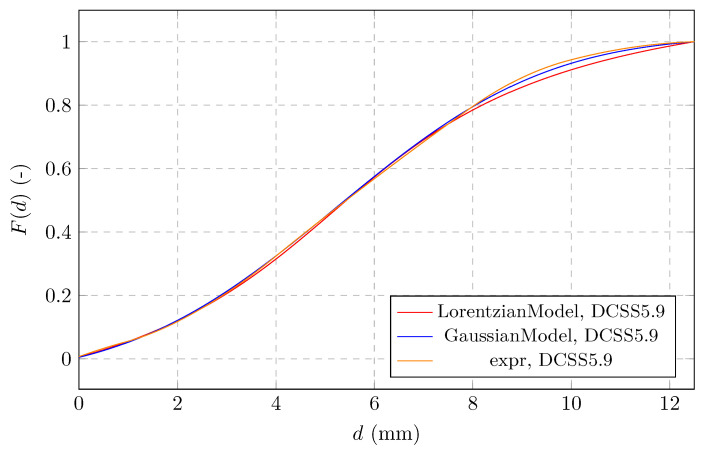
Granulometric distribution.

The model parameters were determined to minimize the empirical results’ fitting error. The least squares method provided by Python’s (version 3.12) library Lmfit was applied. The exemplary results of the model fitting (for selected data) are presented in Figure 7.

In particular, the quality of the fits is now evaluated using the following statistical measures. The coefficient of determination:

(3) $$R^{2}=1-\frac{\sum {\left(y_{i}-{\hat{y}}_{i}\right)}^{2}}{\sum {\left(y_{i}-\bar{y}\right)}^{2}}$$

where $y_{i}$ is the experimental data points, ${\hat{y}}_{i}\;$ is the fitted values, and $\bar{y}$ is the mean of the observed data.

The root mean square error (RMSE):

(4) $$RMSE=\sqrt{\frac{1}{n}\sum {\left(y_{i}-{\hat{y}}_{i}\right)}^{2}}$$

Additionally, the least-squares optimization implemented in the *Lmfit* library minimizes the residual sum of squares (RSS):

(5) $$RSS=\sum_{i\;=\;1}^{n}{\left(y_{i}-{\hat{y}}_{i}\right)}^{2}$$

These criteria provide a quantitative assessment of the agreement between the model and experimental data and ensure proper control over the fitting accuracy.

### 3.2. Test Results Analysis

It is a model of the development of wear of crushing plates. The analysis confirms the feasibility of applying sieve analysis to crusher diagnostics. The beginning of the process is not clearly defined, indicating that the initial stage does not affect the particle size distribution (up to 7.2 mm CSS).

**Figure 8:**
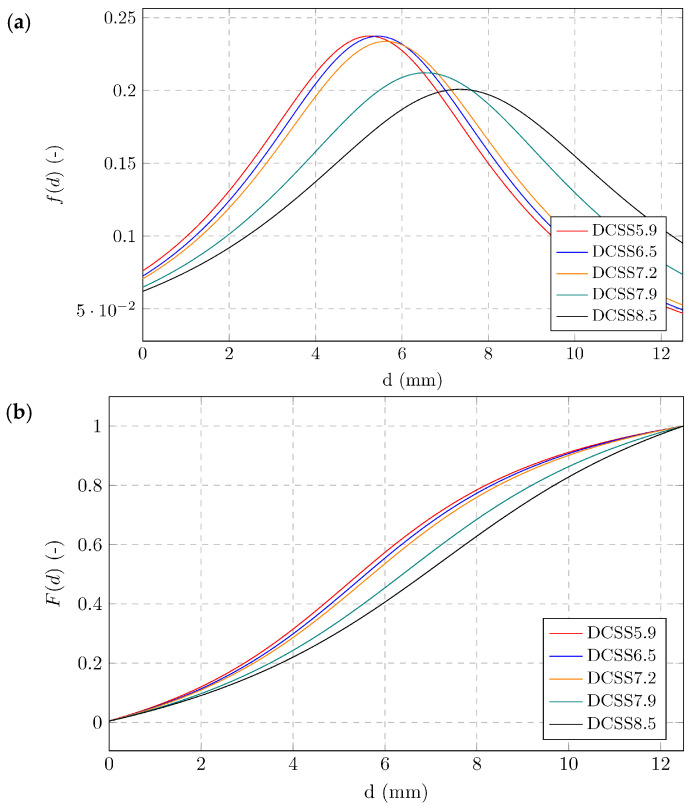
(**a**) Granulometric distribution of dolomite for different values of CSS outlet slots; (**b**) summarized granulometric distribution of dolomite for different values of CSS outlet slots within a range from 5.9 to 8.5 mm. F(d)—passing fraction, f(d)—mass fraction, d—product size.

Results of data fitting for all values of CSS for dolomite are presented in Figure 8, where F(d) denotes the corresponding distribution function, while f(d) corresponds to the mass fraction of the particles.

Parameters of the found distributions are presented in the following Table 3.

**Figure 9:**
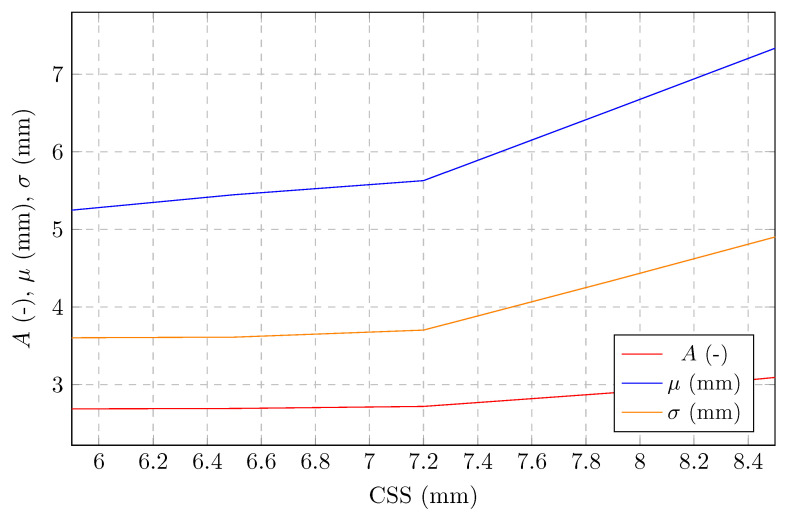
Parameters of the Lorentzian model of product distribution of dolomite for different values of CSS outlet slots within a range from 5.9 to 8.5 mm.

Parameters are presented in Figure 9.

It was found that the grain composition curves of the crushing product can be approximated with high accuracy using the Lorentzian equation. These are three-parameter curves. During research and evaluation of the grain composition of the crusher product (outlet slot), the full grain composition of the material can be recreated with only 3 parameters (Figure 9).

Analysis of the simulation data indicates that the model parameters can be described by linear functions (a regression model). In the case of A and σ parameters, constant values were used. Based on the determined parameters, only the average is visible due to the largest changes in the linear trend; these results are shown in Figure 6. For the mean value, linear approximations were applied.

**Figure 10:**
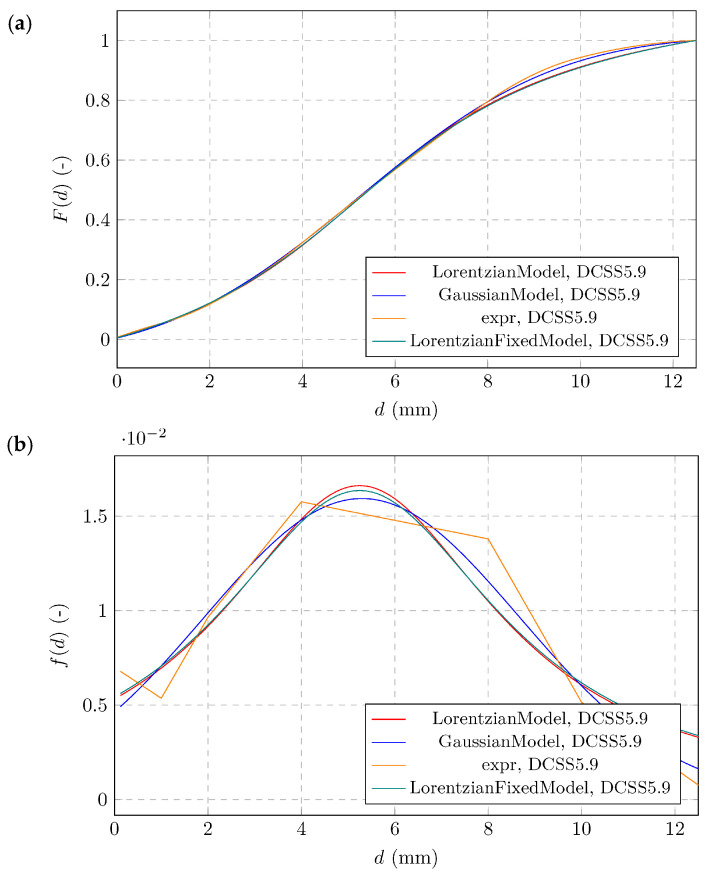
(**a**) CSS 5.9 and 8.5 mm; (**b**) CSS 6.5 and 8.5 mm.

Results presented in Figure 10 show a high level of matching process accuracy (low error). It allows us to replace experimental investigation with the proposed approach.

Figure 10 compares three models with experimental data. The Gaussian and Lorentzian models were fitted with experimental data, as explained earlier. A fixed model was obtained with the simulation data for the Lorentzian model as described. The preliminary evaluation shows that the results are reliable and can be used to assess the operating parameters of the comminution process.

It should be noted that in the initial range (CSS ≤ 7.2 mm), no significant changes in the product’s particle size distribution were observed, which is due to the high consistency of the distributions obtained and the minor differences in the values of the parameters describing the distribution. Lorenzian model has been applied for further investigation because it yields better approximation, despite its higher level of abstraction and lower statistical properties.

This statement is descriptive in nature and based on an analysis of trends in the experimental data. Within the analyzed range, the variability of the results falls within the limits of measurement repeatability, indicating that this parameter has no significant effect on the analyzed product properties.

## 4. Discussion

The results indicate that changing the CSS significantly affects the crushing process and the product particle size distribution, consistent with the literature on jaw crushers. In particular, the observed relationships confirm the importance of the crushing chamber geometry as a key factor in determining process efficiency.

Although the study was conducted on a laboratory scale, with lower energy levels and smaller feed sizes than in industrial conditions, this approach is commonly used to analyze crushing processes in jaw crushers. It enables the identification of fundamental relationships that underpin the interpretation and modeling of processes at an industrial scale [18,55]. At the same time, it should be emphasized that the influence of the crushing chamber geometry, represented by the CSS, on the process behavior and product particle size distribution is fundamental and has been extensively described in the literature. Therefore, despite the limitations imposed by the scale of the experiments, the results obtained may serve as a basis for interpreting analogous phenomena under industrial conditions. However, these limitations point to the need for further verification in larger-scale systems and under conditions closer to the real world. The results refer to the laboratory scale and may differ in industrial conditions due to scale effects [109]. The literature indicates that the transition from laboratory to industrial scale may lead to changes in particle size distribution and crushing process efficiency [110].

The geometry of the crushing chamber is one of the key parameters in crushing [11,31,68,69,77]. The space between the crushing plates affects the efficiency [38,46]. The longer the particles are abraded in the crusher, the smoother their plate surfaces will be. Therefore, the condition of the jaws can either improve or worsen the particle shape characteristics. The geometry of the crushing chamber is not a constant during operation, as wear of the working plates gradually changes the profile of the working surfaces. This phenomenon can result in changes in the effective CSS gap, material flow conditions, and stress distribution in the crushing chamber, which directly affect the process and the product particle size distribution. Due to the lack of direct measurements of the worn plates’ morphology, this aspect was not quantitatively analyzed in this study.

Microscopic characterization (SEM or optical microscopy) of the worn crushing plates was not performed in this study, as the focus was on macroscopic evaluation of the functional effects of wear in relation to the crusher geometry and its operation. Detailed analysis of the wear surface morphology was indicated as a direction for further research.

Sieve analysis can be used to assess the influence of CSS size on the material’s granulometric composition. The gap can be controlled, and its wear can be predicted to the point that exceeding a certain level causes the final product to fail to meet the crushing process specifications.

Sieve analysis samples can serve as a means of monitoring the wear of crushing plates and their impact on the aggregate’s mechanical properties. The presented method can be applied to various rock types and used to assess the quality of the resulting product.

The results of this study highlight the importance of including laboratory crushing in evaluating aggregate shape properties and the effect of the crushing machine’s working chamber on product composition. It is also worth noting that an important parameter for assessing the quality of the crushing process, not considered in this study, is the flakiness index (FI), determined in accordance with EN 933-3 [111].

The proposed method can be applied to jaw crushers and, potentially, to cone crushers; however, this requires further verification. It should be emphasized that jaw and cone crushers differ in their crushing mechanism: jaw crushers operate in cyclic compression, while cone crushers operate in a continuous mode involving compression and shear in a variable chamber geometry [46,112]. Furthermore, in cone crushers, lining wear changes the chamber geometry and the effective CSS gap, affecting crushing conditions and product distribution. Therefore, extending the model to cone crushers requires parameter adaptation and recalibration. This issue is supported by the literature on the effects of geometry and wear on crusher performance [58,59,113,114,115].

This is expected because the outlet slot size determines the product’s grain size, and as plates wear, the distance between the crushing plates will increase.

**Figure 11:**
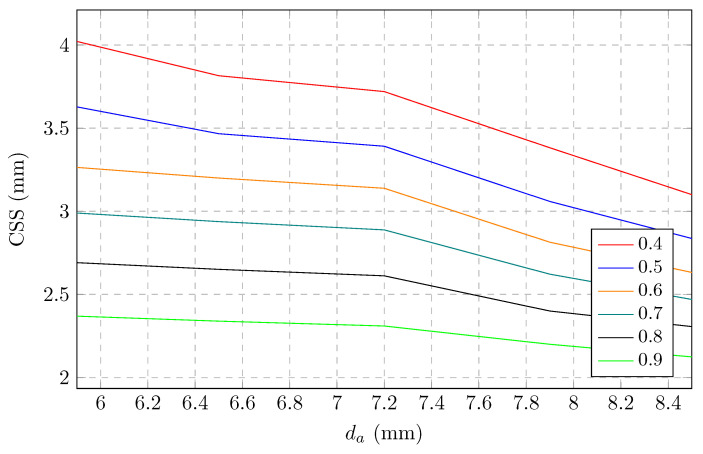
Outlet slot size CSS for various average product sizes across different sizes.

The relationship of the product size (d_a_) to the minimum value of the CSS allows us to see that as the value of CSS increases, the product size also increases, as shown in the figure (Figure 11). The observed changes in grain size d_a_ at level a are associated with jaw wear, as demonstrated in the laboratory study.

The graph in Figure 11 shows the dependence of the maximum diameters of crushed grains on the CSS size. The curves show how the share of grains increases with increasing slot size. Interpretation of the graph indicates that for a slot corresponding to an increase of 9.2% of its value, we are approaching the value from which the share of larger grains starts to increase faster. It can therefore be stated that the granulometric composition of the tested rock will change.

The aforementioned analysis allows us to propose the method of estimation CSS regarding the wear process:

(6) $$CSS_{e}=CSS^{ref}+a\cdot \left(d_{50}-d_{50}^{ref}\right)$$

where $CSS_{e}$—estimated (approximated) CSS, $CSS^{ref}$—CSS with nominal plates and gap, a—increase ratio of CSS and grain size for assumed a level, $d_{50}^{ref}$—average grain size for nominal plates and gap, and $d_{50}$—observed grain size for plates and gap during operation.

Formula (3) can be used to calculate a parameter $w^{\sim }$ that indicates the wear of the crushing plates. The wear coefficient $w^{\sim }$ is an indirect indicator of plate wear, reflecting changes in the CSS and their effects on the crushing process; positive values of $w^{\sim }$ indicate an increase in wear-related changes in the plate profile.

(7) $$w^{\sim }=\left(CSS_{e}-CSS^{ref}\right)/\left(CSS^{ref}\right)$$

where $w^{\sim }$—wear coefficient.

The proposed approach enables observation or examination of the process’s wear coefficient. If it significantly impacts product distribution, the process can be stopped at a convenient time and equipment maintenance performed. However, proposed formula can be used only for initial assessment of the wear on this stage of the research. The primary aim of the application of this coefficient is to assess rate of wear rather than to predict absolute wear values. Further experimental and full validation is needed in further stages.

To avoid the influence of the feed material on the product composition, it is necessary to control the granulometric composition of the feed, for example, by sieving and conducting related sieve analysis. A reference sample of the crushed product should also be prepared, based on which the parameters of the Lorentz distribution model will be determined, as these are essential for the evaluation.

An important element of this work is to demonstrate the potential of parameter variability in the Gaussian and Lorentz distributions for the indirect assessment of jaw crusher jaw wear. The results obtained indicate that selected parameters of the fitted functions—in particular the amplitude parameter (A) and the distribution width parameter (σ)—show a monotonic dependence on the degree of wear of the working plates.

The observed monotonicity of changes is of significant practical importance, as it allows a single model parameter to be used as a diagnostic indicator. In practice, analyzing the particle size distribution of the crushed product and estimating the parameters of the Gaussian or Lorentzian model can be used to assess the degree of jaw wear without requiring direct intervention in the technological system.

As the working plates wear, the CSS value increases, thereby directly affecting the product’s particle size distribution. This change is reflected in the parameters of the adjusted statistical distributions, which confirms the validity of the adopted methodology. The results indicate that parameters A and σ can serve as quantitative measures of working-element wear.

The proposed approach is universal and can be extended to other crushing devices, in particular cone crushers, in which cone wear also alters the geometry of the crushing chamber and modifies the product particle size distribution. However, this requires further experimental research to verify the model’s parameter sensitivity across different operating conditions.

In summary, the use of Gaussian and Lorentzian model parameter analysis is a promising diagnostic tool that enables indirect, quantitative assessment of working element wear based on analysis of the final product of the crushing process.

It should be noted that the present study was conducted with a constant feed particle size range (16–25 mm) to isolate the effect of the closed side setting (CSS). Therefore, the influence of feed particle size on the relationship between CSS and product particle size distribution was not investigated and remains an important direction for future research.

The results indicate that the Lorentzian model parameters *A* and *σ* are related to physical aspects of the crushing process. The parameter A (amplitude) may be interpreted as representing the proportion of particles within the dominant size range, reflecting the intensity of the comminution process and the overall efficiency of the crusher. In turn, the parameter σ (distribution width) describes the spread of particle sizes, indicating the degree of particle size dispersion and the level of material fragmentation.

The observed changes in these parameters with increasing closed-side setting (CSS) and progressive crushing plate wear suggest a direct relationship with the crushing mechanism and the equipment’s operating conditions. In particular, an increase in CSS and wear of crushing elements alters crushing behavior, reflected in changes in the distribution parameters.

Therefore, the parameters A and σ can be considered not only as fitting parameters but also as indirect indicators describing changes occurring in the crushing process, thereby enhancing the physical interpretability of the proposed model.

The present study demonstrates that the Lorentzian model provides an effective and physically interpretable description of the particle size distribution in relation to CSS and crushing plate wear. At the same time, recent developments in machine learning and artificial intelligence indicate a growing potential for their application in modeling complex relationships in crushing processes, including particle size prediction, wear monitoring, and process optimization.

Compared to traditional data-driven approaches, the proposed Lorentzian model offers the advantage of physical interpretability, relatively low computational cost, and clear parameter–process relationships, which are particularly valuable for understanding the underlying mechanisms of crushing. However, machine learning methods, such as random forests or neural networks, may enable the capture of more complex, nonlinear interactions between multiple process variables.

A promising direction for future research is combining physically based models, such as the Lorentzian model, with machine learning algorithms to develop hybrid predictive frameworks. Such approaches could enable real-time monitoring and adaptive control of crushing processes, integrating both interpretability and high predictive performance. Nevertheless, developing such hybrid models requires extensive datasets and dedicated investigations, which fall beyond the scope of the present study.

The proposed Lorentzian-based model can be applied to describe and predict particle size distribution in jaw crushing processes under varying CSS conditions. In practical applications, the model may support the optimization of crusher settings by enabling the estimation of product size distribution and indirect assessment of crushing plate wear based on measurable output data. This provides a useful tool for process monitoring and control in aggregate production.

From an industrial perspective, further optimization of the model could focus on improving its adaptability to varying operational conditions, such as different feed characteristics and equipment configurations. The incorporation of additional variables or extending the model to account for multi-parameter interactions could further enhance its predictive capabilities and practical applicability.

The experimental results demonstrate a clear relationship between CSS, particle size distribution, and model parameters, indicating that the proposed approach can be effectively used to interpret process behavior. This relationship can be used in practice to support decision-making in crusher operations and maintenance planning.

The results indicate the existence of a “non-sensitive” range in which a slight increase in the width of the CSS outlet gap (e.g., by 9.23%) does not result in significant changes in the particle size distribution of the product. This may be related to limitations of the crushing process during the initial phase of working-element wear, when geometric changes in the gap do not yet directly translate into crushing efficiency.

The observed phenomenon constitutes a limitation to the model’s assumption of a linear relationship between CSS and product particle size. Consequently, the model may exhibit reduced prediction accuracy for low CSS values, where this relationship is neither continuous nor linear. Only after exceeding a certain threshold value do changes in CSS clearly affect the product’s particle size distribution.

Taking this effect into account suggests that nonlinear or piecewise models, which better capture the observed transition between the insensitivity region and the region of clear dependence, might be more appropriate. In future studies, it would be worthwhile to determine the upper and lower bounds of this range and assess the impact of this phenomenon on the model’s predictive accuracy.

The width of the CSS insensitivity range may be related to the material’s mechanical properties. The hardness of rocks was not directly investigated in this study; therefore, compressive strength was used as a parameter describing the material’s resistance to fragmentation. A detailed analysis of the effect of hardness requires further research.

An important aspect to consider in future studies is the alignment of aggregate properties obtained under different crusher settings with their intended applications, such as concrete, unbound layers, or bituminous mixtures. This would allow for a more comprehensive assessment of the technological suitability of the aggregates and provide practical guidance for optimizing crushing process parameters.

In this study, the analysis was limited to particle size distribution characteristics, without accounting for parameters describing particle shape, such as roundness or angularity. It should be noted, however, that the morphological characteristics of the grains can provide additional information about the grinding mechanisms and the influence of the machine’s operating parameters. Their quantitative assessment required advanced methods, such as image analysis, and was not included in this study. An analysis of the relationship between particle shape parameters and the CSS value represents a potential direction for further research.

In this study, all experiments were conducted under dry crushing conditions, without considering the effect of material moisture content. This approach allowed for the elimination of additional confounding variables and a clear analysis of the influence of operating parameters, particularly the CSS value.

It should be noted, however, that material moisture content can significantly affect both the crushing mechanisms and the wear process of the crushing plates, including through changes in contact properties, particle adhesion, and the nature of material flow. Therefore, the failure to account for moisture content constitutes a limitation of this study. An analysis of the effect of moisture content on wear and product size distribution requires separate experimental studies and represents a direction for further research.

The proposed wear coefficient model does not explicitly account for material type, as it is based on controlled experimental conditions and focuses on the effects of operating parameters. It should be noted, however, that material properties indirectly affect the model parameters and the characteristics of the particle size distribution.

Consequently, the proposed model is of a general nature; however, its application to different materials requires calibration of the parameters for a specific raw material. The development of an extended model that directly accounts for material properties is a direction for further research and requires systematic comparative analysis for different rock types.

In the studies conducted, the degree of chamber filling was not analyzed as an independent variable. Material feed conditions were kept as constant as possible, ensuring comparable results and minimizing the influence of confounding factors.

It should be noted, however, that the degree of chamber filling can significantly affect the crushing process, the shape of the particle size distribution, and the wear intensity of the working elements. Therefore, the lack of a detailed analysis of this factor constitutes a limitation of this study. Research on variable filling levels, including their quantitative measurement and control, points the way for future work.

The Lorentz model is a three-parameter model that allows simultaneous description of the peak location, width, and amplitude. It should be noted that models with fewer parameters are simpler, but their ability to represent real data is limited. In the analyzed case, the use of a three-parameter model allowed for a high-quality fit, as confirmed by the fit indices.

The choice of model represents a compromise between complexity and data representation accuracy. No signs of overfitting were observed, indicating that the number of parameters used is appropriate for the problem under analysis. A detailed comparison of models with different numbers of parameters may be the subject of further research.

The nominal power of the crusher motor is 1.5 kW; however, actual power consumption during the experiments was not recorded. In this study, the analysis focused on the influence of geometric and operational parameters, in particular the CSS value, on product characteristics and the wear process.

It should be noted, however, that the energy parameters of the process, such as power consumption and crushing energy intensity, can serve as important indicators of crushing efficiency. Taking them into account would allow for a more comprehensive analysis of the relationship between operating conditions, process efficiency, and the wear of working parts. Therefore, extending the research to include an analysis of energy parameters is a direction for further work.

In this study, all experiments were conducted using a single type of crushing plate with a specific geometric profile. This approach enabled limiting the number of variables and clearly analyzing the influence of operating parameters, particularly the CSS value.

It should be noted, however, that the geometry of the working surfaces of the crushing plates can significantly affect the crushing process, the particle size distribution, and the rate of wear. Therefore, the lack of a comparative analysis of different plate profiles constitutes a limitation of this study. Research involving different plate geometries and their impact on the crushing process represents a direction for future work.

The particle size of the feed material was controlled by preparing material with a specific particle size range before feeding it into the crusher, which helped limit the impact of raw material variability on the process. This approach stabilizes operating conditions and minimizes the impact of oversized particles on product composition.

It should be noted, however, that the accuracy of feed particle size control was limited to standard material preparation methods and was not the subject of a detailed quantitative analysis. Under industrial conditions, this control can be implemented through screening or pre-classification systems, enabling more precise process control. An analysis of the impact of particle size control accuracy on crushing efficiency and wear of working parts is the focus of further research.

It should be noted that under actual operating conditions, after passing through the main crushing zone, the material may undergo additional secondary grinding processes associated with interparticle interactions and flow dynamics in the discharge zone. The state of material motion in this zone can influence the final particle size distribution, leading to further fragmentation of finer fractions.

In this study, these phenomena were not analyzed directly, and their influence is treated as part of the complex material flow process. A detailed analysis of these effects requires advanced research methods and provides a direction for further research.

Changing the outlet gap (CSS) can have a significant impact on energy consumption in the crushing process. Smaller CSS values lead to increased interaction intensity between particles and working elements, which may result in higher energy demand while simultaneously promoting the production of finer product fractions and increased wear on the crushing plates.

Conversely, higher CSS values may lead to reduced energy consumption, but at the cost of a lower degree of material fineness. In this study, energy consumption was not directly measured; therefore, the analysis presented is qualitative. A quantitative assessment of the relationship between energy consumption, particle size distribution, and wear of working elements requires further experimental research.

The proposed wear coefficient is based solely on particle size distribution analysis, enabling simple implementation and high physical interpretability. It should be noted, however, that under actual crusher operating conditions, other process signals are also available, such as vibrations, noise, and energy parameters, which can provide additional information on the wear state and the process course.

These parameters were not considered in this study, which constitutes a limitation of the proposed approach. The integration of granulometric data with additional diagnostic signals within multi-parameter fusion models may improve the accuracy of equipment condition assessment and represents an interesting direction for further research.

The proposed wear coefficient can serve as a basis for determining the replacement cycle for crushing plates under industrial conditions. In practice, the timing of replacement of working elements can be determined by exceeding a set threshold value of the wear coefficient or by observing deterioration in product quality, e.g., changes in the grain size distribution or an increase in the average particle size.

This approach links the wear condition of the working elements to the technological requirements of the process. It should be noted, however, that accurate determination of the replacement cycle requires long-term operational data and model calibration under real-world conditions, which are beyond the scope of this work and constitute a direction for further research.

It should be noted that the Lorentzian model used is symmetric and does not directly account for possible asymmetry in the particle size distribution. In real-world conditions, the crushing process can lead to distributions with a certain degree of skewness, resulting from differences in fragmentation mechanisms and material properties.

In the analyzed cases, the observed deviations from the distribution symmetry were small, allowing for the effective use of the Lorentzian model as an approximation. The obtained fits were in good agreement with the experimental data, as confirmed by the fit indices.

For more complex distributions, extended models can be used, such as asymmetric distributions, superposition-based models, or models incorporating skewness parameters. However, their implementation requires more advanced analysis methods and is a direction for further research.

## 5. Conclusions

The developed research method was specifically designed to investigate the influence of changes in the CSS value of a jaw crusher on the granulometric composition of the resulting crushed product and, thereby, on the assessment of crushing plate wear. This project involved conducting experimental studies and mathematical analyses, leading to the development of a mathematical model that describes the relationship between the CSS value and the granulometric composition.

The conducted research introduces a new approach to analyzing the relationship between crusher wear and the quality of the final product, which may contribute to improving the efficiency of monitoring and controlling crushing processes in industrial conditions.

The studies confirmed the initial hypothesis that increasing the CSS gap increases the particle size of the resulting product, with a linear relationship up to a certain point. In particular, it was observed that a relatively small increase in the CSS outlet gap (e.g., by approximately 9.23%) did not result in significant changes in the particle size distribution, indicating the existence of a “non-sensitive” range. Moreover, the study demonstrated that it is possible to safely increase the CSS, thereby extending the operational life of the plates and predicting when an adjustment to the CSS will be necessary. Beyond this range, further increases in CSS lead to noticeable coarsening of the product, confirming the practical importance of monitoring CSS during operation. The configuration of the outlet slot settings used in this study effectively simulates the crushing plate wear process and its impact on the granulometric composition of the product.

Due to its versatility, the proposed method can be used to test various types of crushers and can be adapted for crushing different brittle materials. The model allows for testing crushers with different working chamber sizes. The crushed product can be monitored through sieve analysis or a vision system.

Furthermore, these studies can contribute to optimizing crushed material consumption, which is crucial for minimizing the production costs of the desired aggregate fraction. The efficient use of crushers in aggregate production is also essential due to the associated production costs, process efficiency, carbon footprint, and the energy required for their operation.

This study has certain limitations. The experiments were conducted using a limited number of rock types under dry laboratory conditions, and the influence of factors such as moisture content, feed variability, and industrial-scale operating conditions was not considered. Additionally, the study was restricted to a specific range of CSS values and a laboratory-scale jaw crusher.

Future research should extend the proposed approach to a broader range of materials and operating conditions, including wet crushing and industrial-scale applications. Further work may also include integrating multi-parameter effects, such as rotational speed and jaw kinematics, as well as developing hybrid models combining physical and data-driven approaches to improve prediction accuracy and enable real-time monitoring. The correlation of the mechanics of the crushing process and statistical model should be verified to provide higher level of industrial applicability.

Despite these limitations, the proposed method provides a useful framework for linking crusher operating conditions to product particle size distribution. It offers a basis for further development of advanced monitoring and control strategies in crushing processes.

In further research, it is advisable to expand the scope of the analyses to include actual measurements of crushing plate wear and its direct correlation with changes in CSS values. It would also be advisable to include additional aggregate quality assessment parameters, such as the flatness index (FI), to provide a more comprehensive assessment of product properties. Another important direction for further work is to match the properties of aggregates obtained at different crusher settings to their intended applications (e.g., in concrete, unbound layers, or bituminous mixtures). Furthermore, it is advisable to conduct a more detailed sensitivity analysis of the process parameters and verify the proposed approach in industrial conditions.

In this study, the analysis was conducted based on a limited amount of experimental data obtained under controlled conditions. Although the number of repetitions performed allows for assessing the reproducibility of the results and identifying major trends, it should be noted that the sample size may affect the model’s ability to generalize the obtained relationships.

Increasing the amount of experimental data and covering a wider range of operating conditions and material properties could improve the stability of model parameter estimates and enhance the model’s predictive capability. Therefore, expanding the experimental database represents an important direction for further research.

To expand the application potential of the proposed approach, its implementation in industrial settings as part of crushing process monitoring systems could be considered. An example application scheme involves periodic or continuous collection of product particle size data, fitting a Lorentzian model, and analyzing changes in model parameters as indicators of the wear condition of the crushing plates.

Obtained results are convergent with experimental data. Level of fitting is $R^{2}=0.98$, what allows to state about correct selection of the model. Moreover, it ensures that further investigation of different distributions or performing wider ranges of the experimental works bring better representation of crushing process.

Based on this, it is possible to make operational decisions, such as optimizing the clearance size (CSS) or determining when to replace working parts. This approach allows for linking product quality to the machine’s operating parameters and its wear condition.

At the same time, it should be emphasized that full industrial implementation and quantitative validation of the model require the use of operational data and long-term observations, which constitute the direction of further research.

In industrial practice, grain size distribution analysis is often performed manually, such as by sieve analysis. These methods are characterized by high measurement accuracy but are time-consuming, require operational downtime, and do not allow continuous process monitoring.

The modeling approach proposed in this paper enables the description of grain size distribution and monitoring of changes in process parameters in a more computationally efficient manner. In particular, it can provide a basis for implementing systems that support operational decisions under quasi-continuous conditions.

Additionally, in industrial settings, image-based measurement methods can rapidly and without contact determine product grain size characteristics. Integrating such measurement systems with the proposed modeling approach could enable continuous acquisition of input data and further reduce process control costs.

From an economic perspective, the model, combined with automated measurement methods, can significantly reduce operating costs associated with laboratory analyses and improve operational efficiency by enabling early detection of changes in component wear and by optimizing operating parameters. At the same time, it should be emphasized that the accuracy of the model-based approach depends on the quality of the input data and parameter calibration. A detailed comparative analysis of the accuracy of manual, model-based, and image-based methods, along with their economic efficiency, requires data from industrial conditions and is a direction for further research.

## Figures and Tables

**Table 1:** Comparison of selected studies and identification of the research gap.

Reference	Scope	Approach	Main Findings	Research Gap
Luo et al. (2019) [49]	Wear-resistant materials	Microstructure analysis	Improved wear resistance using TiC-reinforced composites	No link between wear and crushing product characteristics
Machado et al. [47]	Wear mechanisms	Micro-scale tribological tests	Work hardening significantly influences wear behavior	No process-level analysis or PSD consideration
Chen et al. [88]	Jaw plate wear	FEM simulation	Surface modification improves wear resistance	No relation between wear and product granulometry
Terva et al. [16]	Wear–energy relationship	Instrumented crusher experiments	Wear is proportional to crushing work	No PSD analysis
Jiang et al. [31]	Wear evolution in crushers	DEM + Archard model	Wear reduces crusher efficiency and throughput	No direct modeling of PSD or product quality
Machado et al. [51]	Wear modeling	Numerical modeling (Archard-based approach)	Wear depends on contact parameters and modeling assumptions	No application to the crushing process or PSD
Lindqvist & Evertsson [55]	Liner wear in crushers	Experimental + wear modeling	Wear changes the crusher geometry and liner profile	No analysis of the resulting PSD
Machado et al. [50]	Wear in real crushing conditions	Experimental jaw crusher tests	Different wear mechanisms for jaws; higher wear in the stationary jaw	No relationship between wear and PSD or CSS
Coloma et al. [57]	Influence of feed size and geometry	DEM–MBD simulation	Optimized particle size and geometry reduce wear by up to 50%	No PSD or product quality analysis
Quartey et al. [60]	Crusher design influence on wear	Experimental study	Modified jaw design reduces liner wear by ~88%	No PSD or product granulometry analysis
This study	CSS–PSD relationship	Laboratory tests + Lorentzian modeling	Identification of non-sensitive CSS range (~9.23%)	Integrated wear–geometry–PSD analysis

**Table 3:** Fitting parameters of the Lorentzian dolomite model for different values of CSS, fitting parameters of the Gaussian model of dolomite for different values of CSS.

	Parameters of the Lorentzian Model			Parameters of the Gaussian Model		
CSS (mm)	A (-)	μ (mm)	σ (mm)	A (-)	μ (mm)	σ (mm)
5.9	2.69	5.25	3.60	1.91	5.30	3.37
6.5	2.69	5.45	3.61	1.92	5.48	3.38
7.2	2.72	5.63	3.30	1.92	5.66	3.41
7.9	2.89	6.54	4.34	2.00	6.37	3.85
8.5	3.09	7.33	4.90	2.10	7.04	4.27

## Data Availability

The original contributions presented in this study are included in the article; further inquiries can be directed to the corresponding author.
